# *Drosophila*—A Model System for Developmental Biology

**DOI:** 10.3390/jdb12020015

**Published:** 2024-05-21

**Authors:** Nicholas S. Tolwinski

**Affiliations:** 1 Program in Cancer and Stem Cell Biology, Duke-NUS Medical School, Singapore 169857, Singapore; nicholas.tolwinski@duke-nus.edu.sg; 2 Division of Science, Yale-NUS College, Singapore 138527, Singapore

In this Special Issue, titled “*Drosophila*—A Model System for Developmental Biology”, we present a series of articles and reviews looking at the diverse ways that researchers are using the humble fruit fly, also known as the vinegar fly, to tackle the many aspects of development and homeostasis. 

In the more than one hundred years since Thomas Hunt Morgan established *Drosophila* as a major model organism for biological research, the field has contributed a variety of major insights in biology. To this day, *Drosophila* remains one of the most used model organisms in a variety of fields. *Drosophila* have been used to model development and aging, disease and homeostasis, stem cells and differentiation, neurogenesis and neurodegeneration, and many, many other processes. 

In this Special Issue of the *Journal of Developmental Biology*, we provide an overview of the current state of *Drosophila* research and, most importantly, highlight the directions for future invertebrate research. The Special Issue contains eleven review articles, two research papers, a commentary, and an editorial. 

The two research articles cover functions of the Notch signaling pathway during the process of oogenesis and the function of a Dynein adaptor molecule during mitotic and post-mitotic phases. Both papers show novel findings for genes involved in many developmental processes.

The review articles offer a cross-section of research performed in *Drosophila*. Researchers discuss the application of fly research to study the interaction of metabolism and cancer highlighting the use of model organisms to study this fundamental question. As traditional genetic methods have been surpassed by new CRISPR-Cas9-based gene knock in and knock out techniques, *Drosophila* research remains at the forefront, as its genome can now be functionally and easily edited. Further insights are shown from fly research into the function of ubiquitin and small ubiquitin-like modifiers and how these molecules contribute to development, immunity, and cancer. At the fundamental biology level, the transition from maternally provided genes to zygotic transcriptional activation is discussed. An emerging model for stem cell homeostasis, epithelial regeneration, Wnt signaling, and cancer progression is described. Continuing the signaling in development theme, a review describes the conserved signaling pathways that flies use to specify and pattern their appendages. 

Focusing on neurogenesis and neural degeneration, a series of reviews looks at different aspects of these processes. For example, the modeling of human disease in *Drosophila* is a fast, promising, and inexpensive approach for studying neurodegenerative diseases such as Alzheimer’s disease, Parkinson’s disease, Huntington’s Disease, and Amyotrophic Lateral Sclerosis (ALS). A more specific example describes the functions of RNA-binding proteins during both the formation of the nervous system and diseases that affect it, subsequently looking at the complex network of signals that determine the functional integration of neural circuits and the role that cell death plays in this process and at what the role of stem cells is. Of course, apoptosis is not the only way that cells die, so the various ways that cell death can occur are explored. 

The articles and reviews presented in this Special Issue show only a small sample of the great breadth of *Drosophila* research. Finally, I would like to say a big thank you to all the authors and reviewers for their contributions. Without them, ensuring the quality and scientific insight of this Special Issue would not have been possible. I hope these articles will showcase the centrality of model organism work for a variety of researchers and will attract newcomers to collaborate with fly colleagues or join the field in the future.

